# The effect of age of cochlear implantation on vocal characteristics in children

**DOI:** 10.4102/sajcd.v63i1.142

**Published:** 2016-06-17

**Authors:** Kerry Knight, Simone Ducasse, Ashley Coetzee, Jeannie van der Linde, Anel Louw

**Affiliations:** 1 Department of Speech-Language Pathology and Audiology, University of Pretoria, South Africa

## Abstract

**Background:**

Early cochlear implantation aids auditory feedback and supports better communication and self-monitoring of the voice. The objective of this study was to determine whether the age of cochlear implantation has an impact on vocal development in children implanted before age 4.

**Method and procedures:**

The study consisted of 19 participants in total. All implant recipients (experimental group) were 3–5 years post-implantation, including four prelingual (0–2 years) and five perilingual (2–4 years) implant recipients. The control group consisted of 10 children whose hearing was within normal limits between the ages 3–6 years and 10 months, which was compared to the experimental group. Established paediatric norms were used for additional comparison. A questionnaire was used to gather information from each of the participant’s caregivers to determine whether other personal and contextual factors had an impact on voice production. An acoustic analysis was conducted for each participant using the Multi-Dimensional Voice Program of the Computerized Speech Lab.

**Results:**

When the experimental group and the control group were compared, similar results were yielded for fundamental frequency and short-term perturbation (jitter and shimmer). More variability was noted in long-term frequency and amplitude measures, with significantly higher differences, and therefore further outside the norms, in the prelingual group when compared to the perilingual and control groups.

**Conclusion:**

In this study, age of implantation did not impact vocal characteristics. Further research should include larger sample sizes, with participants that are age and gender matched.

## Introduction

An individual’s voice forms the basis of first impressions and is also a means of conveying information effectively (Imhof, Välikoski, Laukkanen & Orlob, 2014). Vocal characteristics influence how a person is conveyed to others, whether it be likeable, attractive, or intelligent (Imhof *et al*., 2014). Voice has a significant influence on the communication partner’s attitude towards the speaker (Seifert *et al*., 2002).

Literature indicates that certain vocal characteristics in individuals with hearing loss can differ greatly in comparison to those with hearing within the normal range (Hocevar-Boltezar, Vatovec, Gros & Zargi, 2005) The age of cochlear implantation impacts vocal maturation of children (Ertmer, Young & Nathani, 2007) as it establishes access to sound which is essential in the maturation of voice. Control of voice pitch, loudness, laryngeal quality, and resonance are inconsistent in individuals with hearing loss who have not received cochlear implants (Lenden & Flipsen, 2007). Children with implants may have difficulties in controlling the pitch and loudness of their voices during sustained phonation, which may result in perceived variation in pitch and loudness (Holler & Campisi, 2010). However, the longer a child has been exposed to auditory feedback, the greater control they have over their voice pitch and loudness (Holler & Campisi, 2010). Early cochlear implantation aids auditory feedback and supports better communication as well as the individual’s ability to monitor their own voice (De Souza, Bevilacqua, Brasolotto & Coelho, 2012). Although it has been reported in the literature, there are few studies which have evaluated the acoustic and perceptual parameters of voice in children who have received cochlear implants (De Souza *et al*., 2012; Hocevar-Boltezar *et al*., 2005; Holler & Campisi, 2010).

Cochlear implants are neuroprosthetic devices that provide hearing sensitivity to individuals with severe to profound hearing loss (Campisi *et al*., 2005). These devices still have limitations in their transfer of auditory data when compared to normal hearing (Uchanski & Geers, 2003). The implant does not give the user normal hearing (Ertmer *et al*., 2007), instead it offers essential auditory feedback on the timing, intensity and frequency of sound (Campisi *et al*., 2005). Studies have emphasised the importance of auditory feedback for voice production, which is essential for vocal maturation (Campisi *et al*., 2005; Seifert *et al*., 2002). It is therefore expected that children implanted at an earlier age and for a longer duration will produce voice nearer to established norms and control groups (Holler & Campisi, 2010). Cochlear implant users receive enough auditory input and feedback to avoid the additional progress of maladaptive speaking strategies such as excess laryngeal tension and negative intraoral air pressure, as well as unusual speech practices such as audible emissions from the nasal cavity (Higgins, McCleary, Carney & Schulte, 2003).

The earlier the child receives a cochlear implant, the greater the auditory experience (Holler & Campisi, 2010). If a child is deprived of auditory stimulation for a long period of time, there will be a marked reduction in neural plasticity (Sharma, Dorman & Spahr, 2002). Therefore, the optimal time for implantation is before auditory deprivation has an impact on the development of the central auditory nervous system, and subsequent speech and language development (Sharma *et al*., 2002).

Children who receive cochlear implants at an earlier age show more progress over time in attaining intelligible conversational speech than children implanted at a later stage (Flipsen, 2008). Those implanted before 3 years of age also perform better in both their expressive and receptive language skills (Anderson *et al*., 2004). It is predicted that a similar trend will occur in terms of vocal development in younger cochlear implant recipients. Another study has shown that children implanted at an early age have improved neuromuscular control of their articulators (De Souza *et al*., 2012). However, no studies have evaluated the impact of age of implantation on the acoustic and perceptual vocal characteristics in children, using defined age groups of children implanted in the prelingual and the perilingual phases (De Souza *et al*., 2012; Holler & Campisi, 2010).

Studies conducted on varying age groups and durations of implantation, that have focused on vocal characteristics of cochlear-implanted children, have shown that prelingually deaf children, who attain a cochlear implant before age 4, have improved control of their pitch and loudness during voice production (Hocevar-Boltezar *et al*., 2005). Younger cochlear implant users achieved vocal maturation sooner in comparison to those who receive implants at a later stage (Ertmer *et al*., 2007). Post-implantation vocal maturation is a process by which prelinguistic children produce increasingly varied and adult-like utterances (Ertmer *et al*., 2007). Should a child be implanted at a later age, vocal maturation may be delayed due to the period of auditory deprivation (Holler & Campisi, 2010). Although the sample size of these studies are small, they indicate that the majority of children showed improvements in vocal development post-implantation, and those implanted at a younger age achieved vocal development earlier than children implanted at a later stage of development (Ertmer *et al*., 2007). Due to the small sample sizes, unreliable and contradicting results have been reported due to the absence of a standardised methodology in the voice assessment and established norms for paediatric voice data (Campisi, Tewfik, Manoukian & Schloss, 2002).

Studies show that cochlear implantation has many benefits for voice production (Coelho, Brasolotto & Bevilacqua, 2015), including improved perturbation measures and phonation control (Campisi *et al*., 2005; Hocevar-Boltezar *et al*., 2005). Poor early development of vocal characteristics in hearing-impaired children can be due to the limited auditory feedback they receive (Coelho *et al*., 2015). The earlier a child is implanted, the more likely they are to develop normal vocal characteristics (Holler & Campisi, 2010) which, in turn, will improve their vocal communication with others. Therefore, the purpose of this study is to compare children who received cochlear implants during the prelingual phase to those implanted during the perilingual phase, as well as to compare the cochlear implant participants to children with normal hearing. The comparison will be conducted to determine whether the age of cochlear implantation, during the prelingual versus the perilingual phase, has an effect on children’s vocal characteristics, specifically vocal control of short- and long-term perturbation and fundamental frequency.

## Method

A cross-sectional experimental design was implemented, including two experimental groups and a control group. It was hypothesised that the group of children implanted during the prelingual phase would have better vocal characteristics than the group of children implanted during the perilingual phase.

### Participants

Purposive sampling was used to select the participants who were prelingual at implantation (Group 1) and those who were perilingual at implantation (Group 2). Convenience sampling was used to select the control group (Group 3). Potential participants for Group 1 and Group 2 were attained from the University of Pretoria Cochlear Implant Unit (UPCIU). These participants were selected based on the following characteristics: age of implantation and the post-implantation duration. Group 1 included participants who received a cochlear implant during the prelingual phase (between the ages 0 and 2 years). Participants in Group 2 were children who received a cochlear implant during the perilingual phase (between the ages 2 and 4 years). All the experimental participants (in Groups 1 and 2) have been implanted for 3–5 years.

The following exclusion criteria were applied during the selection of participants for Group 1 and Group 2: (1) failure to complete the voicing task, typically because of young age and (2) the presence of any additional/multiple disabilities that may have an impact on voice production. These disabilities included auditory neuropathy, chronic sinusitis, neurological disorders or any other disabilities or disorders that impact voice production. No participants were excluded from the study as they were all able to complete the voicing task and were selected based on the absence of any of the above mentioned disabilities. Uni- and bilaterally implanted children were not separated into two groups due to the small sample size; however, all experimental participants had bilateral amplification (either two implants, or an implant and a hearing aid). As the cochlear implant recipients who complied with the inclusion criteria were scarce, the groups were not stratified according to gender.

Group 3 consisted of typically developing children with no diagnosed voice disorder. Participants were selected based on their age (3–7 years), the same age range as the selected implanted participants. Finally, the participants were required to be physically well on the day of the assessment, as throat or sinus infections would affect voice quality.

In total, 19 participants were included in the study. The prelingual group consisted of four bilateral implanted participants. The perilingual group had five participants, three with bilateral implants and two with unilateral implants and hearing aids on the non-implanted ears. The control group had 10 participants. Of the 19 participants, 6 (32%) had parents who smoke. More female participants (75%, *n* = 3) were included in the prelingual group, and the mean age of the participants was 4.94 years (SD = 0.98). The perilingual group consisted of only male participants (100%, *n* = 5), and the mean age of the participants was 6.73 years (SD = 1.01). The control group had an even distribution of gender, female (50%, *n* = 5) and male (50%, *n* = 5), with a mean age of 4.28 years (SD = 1.14).

### Material and apparatus

A background information questionnaire was completed by the parent or guardian of the participant. Questions regarding the participant’s biographical information, health and medical history, as well as any environmental factors that may have an impact on the participant’s voice were included in the questionnaire. The questionnaire consisted of 20 closed-ended and open-ended questions. A published questionnaire (Shipley & McAfee 2009) was used as a guide in developing the questionnaire.

A voice sample was obtained from each participant. The voice samples were analysed using the Multi-Dimensional Voice Program (MDVP) of the Computerized Speech Lab (CSL) (model 4500; KayPENTAX). The MDVP analyses the voice sample and provides acoustic information to determine vocal parameters. Table 1 summarises the parameters evaluated.

**TABLE 1:** Vocal parameters.

Parameters	Description
Fundamental frequency (Fo/Hz/)	Fo is the perceived pitch of the voice sample (Skuk & Schweinberger, 2014)
**Frequency perturbation measurements**	
Jitter per cent (Jitt /%/)	Jitt /%/ evaluates the very short-term variability of the voice sample’s pitch period (Hocevar-Boltezar *et al*., 2005)
Fundamental frequency coefficient variation (vFo /%/)	Refers to voice pitch (Holler & Campisi, 2010). It is the long-term fundamental frequency variation (Campisi *et al*., 2005)
**Amplitude perturbation measurements**	
Shimmer per cent (Shim /%/)	Shim /%/ evaluates the very short-term variability of the voice sample’s peak-to-peak amplitude (loudness) (Hocevar-Boltezar *et al*., 2005)
Peak-to-peak amplitude coefficient of variation (vAm /%/)	Refers to voice intensity (Holler & Campisi, 2010). It is the peak amplitude variation (Campisi *et al*., 2005)

### Procedures

Ethical clearance was obtained from the Research and Ethics Committee of the Department of Speech-Language Pathology and Audiology, University of Pretoria, and permission was given by the UPCIU to access their database to identify potential participants to form part of the experimental group.

Parents or guardians of potential participants were fully informed of the purpose of the study and informed consent was obtained. Verbal assent was obtained from the participants. The confidentiality of all participants was ensured throughout the study process.

The assessment was conducted at the Department of Speech-Language Pathology and Audiology at the University of Pretoria. First, the questionnaire to gather biographical and background information of the participants was conducted in the form of an interview with the parent or guardian. Explanations were provided where necessary to ensure understanding. After the questionnaire was completed the voice recording was made. Participants were seated in a soundproof room. A standardised microphone was placed in front of them at an off-axis position of 45° and a constant mouth-to-microphone fixed distance of 10 cm. Participants were instructed to produce the vowel /*a*/ for three seconds using a comfortable pitch and volume. This was repeated three times. The best and most consistently produced sample of the three trials was used for analysis. The phoneme /*a*/ was chosen because it is a steady-state vowel and is easy for children to reproduce. The voice samples were analysed using the MDVP of the CSL (model 4500; KayPENTAX, Lincoln Park, New Jersey).

### Data analysis

All statistical analyses were performed using the commercially available software Statistical Analysis System (SAS®) version 9.3 (SAS Institute Inc., 2011) which was run using Microsoft® Windows® on a personal computer. The study aimed to determine whether the age of cochlear implantation had an effect on the vocal characteristics in children, implying a comparison between Group 1 and Group 2, which was the primary objective of the study and the analysis. Results obtained from Groups 1 and 2 were analysed descriptively because of the small sample size. Nevertheless a *t*-test (Gosset, 1908) was performed to compare mean values of vocal parameters in cochlear implant users varying in age of implantation with previously published paediatric norms based on a sample of an American population (Campisi *et al*., 2002). The normative database was made up of 100 control participants (50 boys and 50 girls) between the ages 4 and 18 years (Campisi *et al*., 2002). A comparison was also made between mean values of vocal parameters of the cochlear implant users and the control group of normal hearing participants. Normality of the underlying distributions (required for *t*-tests) could not be verified but could be substantiated. Due to the small sample sizes, all the results from the statistical analysis should be interpreted as descriptive or preliminary results and not as conclusive, which may guide future research and clinical practice.

## Results

In order to determine whether the age of implantation had an impact on vocal characteristics, specifically vocal control of children with cochlear implants, a number of acoustic measures were evaluated. The mean values of jitter, shimmer, fundamental frequency, fundamental frequency variation or vFo and amplitude variation or vAm were compared between the two experimental groups, the control group, as well as the established normative data (Campisi *et al*., 2002). The mean values and standard deviations of vocal parameters between the three groups as well as the paediatric norms are presented in Table 2.

**TABLE 2:** Acoustic outcomes of cochlear implantation users (prelingual and perilingual), the controls and the paediatric norms for vocal parameters.

Variable	Frequency	Jitter	Shimmer	vFo	vAm
Prelingual (*n* = 4)	304.53 (SD±29.44)	1.71 (SD±2.13)	3.56 (SD±1.73)	6.23 (SD±4.53)	36.30 (SD±11.30)
Perilingual (*n* = 5)	265.82 (SD±26.66)	1.79 (SD±1.09)	5.10 (SD±2.23)	4.43 (SD±2.43)	22.92 (SD±8.00)
Control (*n* = 10)	297.74 (SD±58.01)	1.23 (SD±0.45)	5.72 (SD±1.93)	2.80 (SD±0.83)	22.37 (SD±7.33)
Prelingual and perilingual (*n* = 9)	283.02 (SD±33.11)	1.76 (SD±1.52)	4.41 (SD±2.07)	5.23 (SD±3.40)	28.87 (SD±11.38)
Paediatric norms (*n* = 100)†	279.05	1.24	3.35	1.75	15.1

† Normative data was obtained from Campisi *et al*. (2002).

The perilingual group had a substantially lower fundamental frequency (265.82 Hz) when compared to the prelingual group (304.53 Hz). When the experimental groups were compared to control group participants of similar ages, all implanted participants had higher long-term frequency (vFo) (prelingual vFo = 6.23, SD = 4.53; perilingual vFo = 4.43. SD = 2.43) and amplitude (vAm) (prelingual vAm = 36.30, SD = 11.30; perilingual vAm = 22.93, SD = 8) perturbation measures than the control group (control vFo = 2.80, SD = 0.83; control vAm = 22.37, SD = 7.33). The short-term perturbation scores of three of the four participants in the prelingual group were lower (prelingual jitter = 1.71, SD = 2.13; prelingual shimmer = 3.56, SD = 1.73) than those obtained by the paired similar-aged control group participant (control jitter = 1.23, SD = 0.45; control shimmer = 5.72, SD = 1.93). When comparing the short-term perturbations of the perilingual participants to a paired similar-aged control group participant, three of the five perilingual participants had lower jitter percentages and all five had lower shimmer percentages (perilingual jitter = 1.79, SD = 1.09; perilingual shimmer = 5.10, SD = 2.23). These findings indicate that, in general, implant participants had lower short-term perturbations (jitter and shimmer) and higher long-term frequency (vFo) and amplitude (vAm) perturbations than similar-aged control group participants. Variances in fundamental frequency were consistent with gender difference.

The performance outcomes of the prelingual and the perilingual groups were combined and were compared with the results of the control group for the purpose of comparing children with and without cochlear implantation (CI). When compared, similar results were yielded for fundamental frequency (pre- and perilingual CI = 283.02 Hz; control = 297.74 Hz), and short-term perturbation measures including jitter (prelingual and perilingual CI = 1.76%; control = 1.23%) and shimmer (prelingual and perilingual CI = 4.41%; control = 5.72%). The implanted group had slightly higher long-term frequency (prelingual and perilingual CI = 5.23%; control = 2.80%) and long-term amplitude (prelingual and perilingual CI = 28.87%; control = 22.37%) perturbation scores when compared to the control group. The vocal parameters of the various groups are compared in Table 3.

**TABLE 3:** Differences in vocal parameter outcomes between groups.

Variable	Frequency	Jitter	Shimmer	vFo	vAm
Prelingual and perilingual CI	0.240	0.920	0.261	0.279	0.030*
Prelingual CI and control	0.811	0.479	0.082	0.028*	0.013*
Perilingual CI vs. control	0.235	0.377	0.573	0.230	0.906
CI (prelingual and perilingual) and control	0.513	0.310	0.172	0.067	0.153
Prelingual and paediatric norms	0.182	0.688	0.827	0.143	0.033*
Perilingual and paediatric norms	0.329	0.322	0.155	0.069	0.094
Control and paediatric norms	0.335	0.943	0.004*	0.003*	0.012*

CI, cochlear implantation.

* Statistically significant when *p* ≤ 0.05.

Children implanted prelingually had a significantly higher long-term amplitude perturbation (vAm; *p* = 0.030) in comparison to those implanted perilingually. The children who were prelingual at implantation had significantly higher long-term frequency (vFo; *p* = 0.028) and long-term amplitude (vAm; *p* = 0.013) perturbation scores when compared to the control group. The long-term amplitude perturbation of the prelingual group was significantly higher (vAm; *p* = 0.033) than the paediatric norms. Several parameters were significantly higher in the control group when compared to the paediatric norms, including short-term amplitude perturbation (shimmer; *p* = 0.004), long-term frequency (vFo; *p* = 0.003), and long-term amplitude (vAm; *p* = 0.012) perturbation.

No statistically significant differences were found between the performance variables of CI (prelingual and perilingual) combined and the control; this, therefore, indicated that the vocal characteristics of children with and without implantation did not differ significantly.

## Discussion

The values of fundamental frequency, jitter and shimmer revealed no significant differences between the three groups, except the shimmer of the control group when compared to the paediatric norms (*p* = 0.004). The short-term variation of frequency (jitter) and amplitude (shimmer) were within the normal range, which according to Holler and Campisi (2010), is expected when a population does not have a primary laryngeal pathology. Interestingly, more variability in long-term frequency and amplitude in vocal productions were noted in the control group when compared to the paediatric norms. However, the age of the control group ranged from 3.00 to 6.83 years (*n* = 10), whereas the paediatric norm age ranged from 4–18 years (*n* = 100). Therefore, a difference in vocal performance can be expected due to maturation. A previous study established a normative paediatric acoustic database, developing a growth chart for both age- and gender-based vocal maturation (Maturo *et al*., 2012). The study by Maturo *et al*. (2012) included similar norms for fundamental frequency, jitter and shimmer, as well as other acoustic parameters such as noise to harmonic ratio and mean peak airway pressure. However, norms were not established for long-term variation, and therefore, these norms were not used for comparative purposes in this study. The paediatric norms by Campisi *et al*. (2002) used in this study were similar to those published in Maturo *et al*. (2012). Thus, comparisons yielded similar results.

The perilingual group, of which all the participants were male, had a lower mean fundamental frequency than the prelingual group, although the difference was not significant. These findings are consistent with previous research that indicated that there may be a slight or no difference in fundamental frequency between boys and girls under 12 years (Perry, Ohde & Ashmead, 2001).

Findings indicated that the prelingual group differed significantly from the perilingual group (*p* = 0.030), the control group (*p* = 0.013) as well as the paediatric norms (*p* = 0.033) with significantly higher long-term amplitude variation (vAm). The prelingual group also differed significantly from the control group (*p* = 0.028) with substantially higher long-term frequency variation (vFo). These findings are similar to the study by Campisi *et al*. (2005) who also stated that long-term variations remain significantly higher in children with cochlear implants. Yet the perilingual group performed similarly to the control group without any significant differences in vocal performance. The perilingual participants received their cochlear implants at an older age, and previous research suggests that the earlier a child is implanted the sooner they achieve vocal maturation (Ertmer *et al*., 2007). However, in this study the group implanted perilingually (i.e. an older age) produced vocal productions closer to the control group and paediatric norms when compared to the performance of the group implanted prelingually (i.e. at a younger age).

Similar results were found between the vocal characteristics of the prelingual and perilingual groups combined when compared to the control group. The long-term variations were slightly higher in the implant group than in the control group of normal-hearing children, however the differences were not significant. Therefore, these results, in contradiction to previous studies (Ertmer *et al*., 2007; Flipsen, 2008; Holler & Campisi, 2010; Sharma *et al*., 2002), indicate that the vocal characteristics, more specifically vocal control of short- and long-term perturbation and fundamental frequency, of children with implantation did not differ significantly from normal-hearing children.

The results of this study suggest that children, who are implanted at a later stage, before age 4, are not disadvantaged in terms of their vocal development. These findings do not coincide with the initial hypothesis. However, a previous study had similar results, in which children implanted earlier than 4 years of age had better control of their voice at a faster rate and to a larger extent than those who were implanted at a later stage of development (Hocevar-Boltezar *et al*., 2005). In South Africa, the average age of implantation is around 43 months or three and a half years of age (Le Roux, Swanepoel, Louw, Vinck & Tshifularo, 2015). All but one of the children in the study sample were implanted before the average age of implantation for South African children which implies vocal maturation closer to that of normal-hearing children. The study by Hocevar-Boltezar *et al*. (2005) indicated that children implanted before age 4 still achieve positive outcomes in terms of vocal control over time in comparison to children implanted later. This may explain why no significant differences were found between the results for the prelingual and perilingual groups, as the age of implantation did not have an impact on their vocal developmental outcomes. However, due to the small sample size in this study, the results can be seen as descriptive of the specific population.

The main limitation of the study was the sample sizes and as a result, only descriptive methods could be used to interpret the data collected. However, the overall size of the implanted population from which prospective participants could be selected is small. In future, researchers should obtain a larger population in order to make use of inferential statistics for more conclusive results. The gender distribution across the groups was not equal; so direct gender comparisons were not made and possible gender differences were not identified. In future research, participants in all groups should be both gender- and age-matched. It is recommended that the effect of chronological age on vocal maturation be explored for any possible correlation. The development of more age-specific paediatric norms, by including children from 3 years of age and including long-term perturbation parameters, is recommended to establish a more accurate normative database for comparison.

## Conclusion

This study aimed to compare vocal characteristics of children who received cochlear implants during the prelingual phase (0–2 years) and the perilingual phase (2–4 years). Implant groups were compared with both the control group and previously established paediatric norms. Results showed that when cochlear implant and control participants were compared, similar results were yielded for fundamental frequency and short-term perturbation measures (jitter and shimmer). More variability in the prelingual group was noted in long-term frequency and amplitude perturbation, when the prelingual group was compared with the perilingual and control groups. The age of implantation did not have an effect on vocal control, when the prelingual and perilingual CI groups were compared. Vocal control of the implanted children was also similar to children with normal hearing. It therefore appears that, if no other voice problems are present, vocal control may not need to be targeted during intervention for CI recipients. Differences found between the paediatric norms and the control group were as a result of the larger age range used in the establishment of the paediatric norms. The development of more age-specific paediatric norms, both worldwide and specifically for the South African population, is recommended in order to have a more accurate normative database for comparison. Results were interpreted using descriptive methods due to the small sample size. In future, researchers should obtain a larger population with participants that are both gender- and age-matched in order to make more direct comparisons. Future researchers should separate the experimental group into uni- and bilaterally implanted participants to determine whether a relationship exists between the type of implantation and vocal development. It is recommended that the effect of chronological age on vocal maturation be explored for any possible correlation.
